# Mr. Davies's Case of Poison by Arsenic

**Published:** 1812-11

**Authors:** D. H. Davies

**Affiliations:** No. 27, Carburton-street, Fitzroy-square


Medical and Physical Journal.

5 OF VOL. XXVIII.] NOVEMBER, 1812. [NO. 165.

### *To the Editors of the Medical and Physical Journal.*

GENTLEMEN,

IF the following Cases of Poison by Arsenic, and consequent Remarks, meet with your approbation, I shall feel obliged by your inserting them in your widely-circulated Journal, as I am unacquainted with any work so likely to promulgate facts connected with medicine on so extended a scale.

Early on Thursday morning, the 9th of July, about eight o'clock, a mixture, supposed to contain cream of tartar, sulphur, and treacle, was given to four children belonging to J. Cooper, a painter, residing at No. 6, George-street, Portland Chapel. Their ages were nearly as follow: the eldest about nine, the next about seven, the third about five, and the youngest about three, years old. They all took in the morning nearly the same quantity, about a tea-spoon full and a half. From the account I gathered of persons who had an opportunity of seeing them previous to my being sent for, the first symptom which occurred was sickness at stomach every three or four minutes, attended with pain and heat. In the course of a few hours after these symptoms made their appearance, an account of the occurrence was sent to a chemist in the neighbourhood, stating the circumstance, who advised, without seeing the children, large quantities of warm water to be administered frequently. This mode of treatment, however, gave no visible signs of relief, the act of vomiting still continuing at intervals, attended with pain at the stomach.

At about a quarter to eight in the evening, I was requested to visit the children. The only intimation I had, on entering the apartment where the whole four lay, was, that such a mixture as I have mentioned before had been taken by each of the children, not the slightest suspicion having been thrown out by any one present that any thing of a deleterious quality had been given; consequently I felt more surprised at the unusual symptoms that had made their appearance. The whole four of the children had been affected more or less in the same way, by experiencing almost incessant sickness; which, I ought to mention, had come within an hour or two after taking the mixture. When I first saw them, I think the whole four were stretched about different parts of the same bed; one, however, in particular, seemed to be suffering much more than the others, which was the youngest but one, to whom I paid more particular attention at the time. This was the girl, the rest were boys. I questioned her as to the feelings she experienced, and she told me that she felt as if some living animal was constantly gnawing at her stomach, at the same time feeling a sensation of burning heat.

From the history of the symptoms, I suspected some very serious mistake had been committed; but, wishing as a first object to rid the stomach of as much of the pernicious substance as possible, I ordered a strong emetic of the Zinc. Vitr. to be given to each, according to their ages. As the girl appeared evidently to be the worst, the puke was given to her *immediately*, but did not take any effect, though made sufficiently powerful. In the course of ten minutes after she had taken it, I was informed by the by-standers, the countenance was much altered, the circumference about the eyes looked darker than usual, and the mouth was observed to be in the same state; and, in about half an hour from the time of her taking the vomit, she died apparently under the influence of convulsion. This child died just twelve hours after taking the fatal mixture; my attention was now wholly directed to the welfare of the other children. As I stated above, all of them had a strong vomit administered, which, with the exception of the child who died almost immediately after taking it, acted in a very powerful way, by making them reach several times. The substance thrown off the stomach had apparently the consistence and appearance of pus, mixed with adventitious matter. It may not be improper here to mention, that the youngest of the children was excessively sick after taking this mixture in the morning, more so than any of the others, and in a very short time after the taking of it. This, I conceive, was a very fortunate occurrence, as I have no doubt a considerable quantity of the poisonous substance was thrown off the stomach. Inflammation was at this time going on in a very rapid degree in both the other children; the pulse was amazingly high, amounting to 110 strokes in a minute; skin hot; mouth clammy and parched; and a good deal of tenderness about the scrobiculus cordis and surface of the bowels.

Having cleared away as much as possible the contents of the stomach, it was resolved to give three grains of calomel every two hours, following it by repeated doses of a solution of Epsom salts, with the view of diminishing inflammation, and carrying the remains of the pernicious substance through the bowels; and, in addition to this plan of treatment, an oily oily clyster was administered *occasionally* to both of the children.

My curiosity was now excited to know the cause of this dreadful occurrence, by ascertaining what this mixture contained; not the slightest hint, as I mentioned before, having been suggested by any one that arsenic had been given. I therefore sent for the bason containing the remains of the mixture which the children had not taken; and, by the assistance of a friend, we discovered satisfactorily that evening that it contained arsenic; but, as several and repeated tests ought always to be employed to ascertain a fact, I shall detail at the conclusion of the paper a concise account of the different processes that were instituted to prove the identity of the mineral, and place the fact beyond a doubt.

Early next morning, the 10th, I visited the children. The youngest apparently suffered nothing from what he had taken, but it must be recollected he almost instantly rejected the mixture after taking it. This circumstance, I think, sufficiently accounts for his being exempted from the symptoms it produced on the others. Every symptom of inflammation had increased since last night in both the other children. The pulse this morning was amazingly rapid and full; but in one the poison evidently had been taken up with greater avidity by the circulation, and the morbid effects of it were considerably more apparent. One circumstance attending more particularly the case of this child, may be worth notice, which is, that, for intervals of an hour or two, the greatest languor and prostration of strength would prevail, and the pulse for that time (though not generally) would be hardly perceptible; and in an hour afterwards every symptom of increased strength and animation would return, and the pulse would rise in strength and fullness above its previous standard.

At this period, the active state of inflammation was going on (if I may use the term) in the regular way in the other child, that is, no intermissions of any consequence, or other symptoms of immediate danger, were observable; it was therefore advised by Dr. Outram, who attended with myself, to take a quantity of blood, about eight ounces, from the arm, and apply some leeches to the surface of the abdomen. This was done, and a large blister applied.

In the afternoon I saw the children again; the youngest of the two was apparently much worse, the eldest, whom I bled, was somewhat better than in the morning, but still in great danger. I left the youngest with a full conviction that he would not survive the night. My fears were verified, for, late in the evening, I received a message that the youngest of the two was dead. No particular appearances were observable attendant on this child's death; he died, I am informed, perfectly tranquil. It is here necessary to say I obtained permission to have the bodies inspected, which was done with considerable accuracy by Mr. Blagdon, a friend of mine, who made the most minute investigation with respect to morbid appearances; but, as I conceive statements of this nature ought to be as distinct and explicit as possible, I shall subjoin the history of the dissections, with an account of the chemical analysis of the mixture given to the children, at the close of the paper.

I have now only to add, that the treatment adopted in the case of the surviving child, was by giving a saline aperient mixture, rather alkaline, with the frequent administration of an emollient laxative clyster. This plan, I am happy to say, succeeded beyond the most sanguine expectations of every one present; and, in order to prevent prolixity in the statement of the cure and circumstances attendant, I have only to observe, the child recovered from the baneful effects of this pernicious mineral, and is now perfectly well. The only singular circumstance attendant on the cure, and which I think I am justifiable in noticing, was a vesicular eruption which appeared only about the mouth and the circumference of the eyes, on the fourth day after taking the poison. This had every appearance of the maturation of the small-pox in its early stages; in a day or two it formed a crustaceous appearance, and gradually died away.

From the result of the above cases, I feel authorised, as far as my own observation will allow me to judge, to suggest the following remarks:—First, that, had any medical practitioner been called in within an hour or two after taking the poison, the whole of the children might have been saved. Some time must ever elapse between mineral poison being received into the stomach, and its being taken into the circulation; consequently, if the contents of the stomach are early and completely evacuated by the effects of powerful emetics, the fatal consequences must be prevented. The next natural inference to be drawn from the history of the above cases is, that, when inflammation is but recently commencing in the system, from the absorption of the mineral, and the morbid symptoms have not reached to any alarming height, that blood-letting constitutionally and locally is one of the first and most immediate remedies pointed out. The fortunate issue of the case of the surviving child, though not perhaps a conclusive, yet I think is a very strong presumptive, proof of its efficacy in these unfortunate cases. I had previously previously witnessed one case at Nottingham, in a young woman who had swallowed a quantity of the white oxyde of arsenic, who was apparently cured by the early use of venesection, when symptoms of inflammation had just began to make their appearance, which may, perhaps, make me feel more confident of its success; but I must confess it appears to me to be the most promising means, together with evacuating the bowels by mild injections.

*Analyses of the Mixture, part of which was given to the Children.*—I conceived the most conclusive way of proving the identity of arsenic in the mixture, was by first decanting it in different waters till the most ponderous ingredient subsided to the bottom. This was carefully selected, and, by the kind assistance of Mr. Selway, of Upper Mary-le-bone-street, the following conclusive tests were effected.

1. Three grains of the white oxyde of arsenic were dissolved in half an ounce of water, with one drop of liquor ammon.; one drop of a solution of arg. nitrat. was then added to the solution, and a yellow precipitate immediately took place. The same experiment was tried with the decanted substance, and the same result took place.

2. One grain of arsenic was next dissolved with three grains of Kali ppt. in half an ounce of water; and, with the addition of two or three drops of the solution of arg. nitr. a yellow precipitate was immediately found. The same test was tried with the decanted powder, and exactly the same result followed.

Lastly. In order to reduce it to its *metallic* state, one grain of white oxyde of arsenic was mixed with three grains of black flux, and placed in a glass tube, and exposed to heat. The metallic crust sublimed to the sides of the vessel, and gave out an alliaceous smell. The same experiment was tried with the decanted powder, with completely an analogous result.

These several tests and experiments, made in a very cautious manner, place it beyond a supposition that the fatal ingredient in the mixture given to these unfortunate children, was the oxyde of arsenic.

*Of the Appearances on Dissection.*—On examining the stomach of the child who died first, little mark of inflammation was observable on the external surface. The duodenum extremity of the larger curvature exhibited more marks of the effect of inflammation than any other part of the external coat of the stomach; but, on examining the internal surface, the villous coat was found to have been in a very high state of inflammation; but, on dissecting away this coat, the marks of inflammation were found to extend no farther. The colon colon was very much corrugated, particularly about the arch. The duodenum was slightly inflamed, and the surface of the whole of the small intestines was apparently more vascular than usual. The lungs were found not to be in a perfectly healthy state, but which may in a great measure be accounted for, the child having previously labored under hooping-cough, and therefore I think the morbid appearances in that viscus are not attributable in any degree to the effects of the arsenic taken.

In the case of the second child who died, the mineral had been more slow in its operation, and, though not so violent in its effects as in the instance of the other child, yet, having had more time for its operation previous to death, the morbid appearances were more marked and general. The external surface of the stomach in this child was slightly inflamed: on opening it, two or three ounces of coffee-colored fluid was evacuated. The villous coat was most highly inflamed, considerably more so than in the other child. The whole of the internal surface of the small intestines was highly inflamed, particularly the rectum. The internal surface of the bladder exhibited an appearance much whiter than usual. The head was opened in this case, but no particular appearances were observable, except rather an unusual turgescence on the external surface of the brain, and the plexus choroides being remarkably vascular.

Not having taken my notes of the above cases on the spot, I have been compelled to commit them to paper rather in an irregular way; but, as my sole end and aim is to give the facts publicity, I must claim indulgence from your readers as to the order in which they are related.

I am, Gentlemen,

*No.* 27, *Carburton-street,* Your obedient Servant,

*Fitzroy-square.* D. H. DAVIES.

